# Line-field confocal optical coherence tomography imaging findings of scalp psoriasis

**DOI:** 10.1016/j.jdcr.2023.06.050

**Published:** 2023-07-26

**Authors:** Thu M. Truong, Gaurav N. Pathak, Babar K. Rao

**Affiliations:** a Rao Dermatology, Atlantic Highlands, New Jersey; b Department of Dermatology, Rutgers Robert Wood Johnson Medical School, New Brunswick, New Jersey; c Department of Dermatology, Weill Cornell Medicine, New York, New York

**Keywords:** confocal imaging, LC-OCT, line-field confocal optical coherence tomography, psoriasis, scalp psoriasis, spongiotic dermatitis

## Clinical presentation

**Fig 1:**
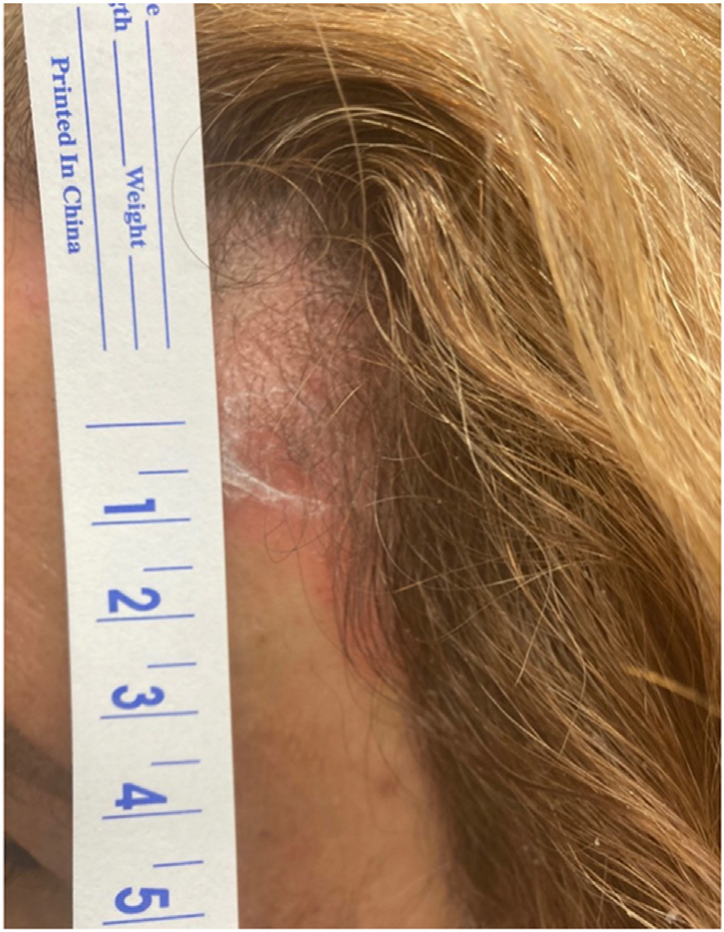
Clinical image of a red scaly plaque.

A 64-year-old woman presented to the clinic with two pink, scaly plaques with excoriations on the right side of the temple and the left side of the abdomen. The initial clinical suspicion was eczema, and the patient was instructed to apply hydrocortisone 2.5% cream to the area. There was no resolution after 2 months of treatment, and the patient was imaged with line-field confocal optical coherence tomography (LC-OCT), and a shave tangential biopsy was also performed (Fig 1).

## LC-OCT appearance

**Fig 2:**
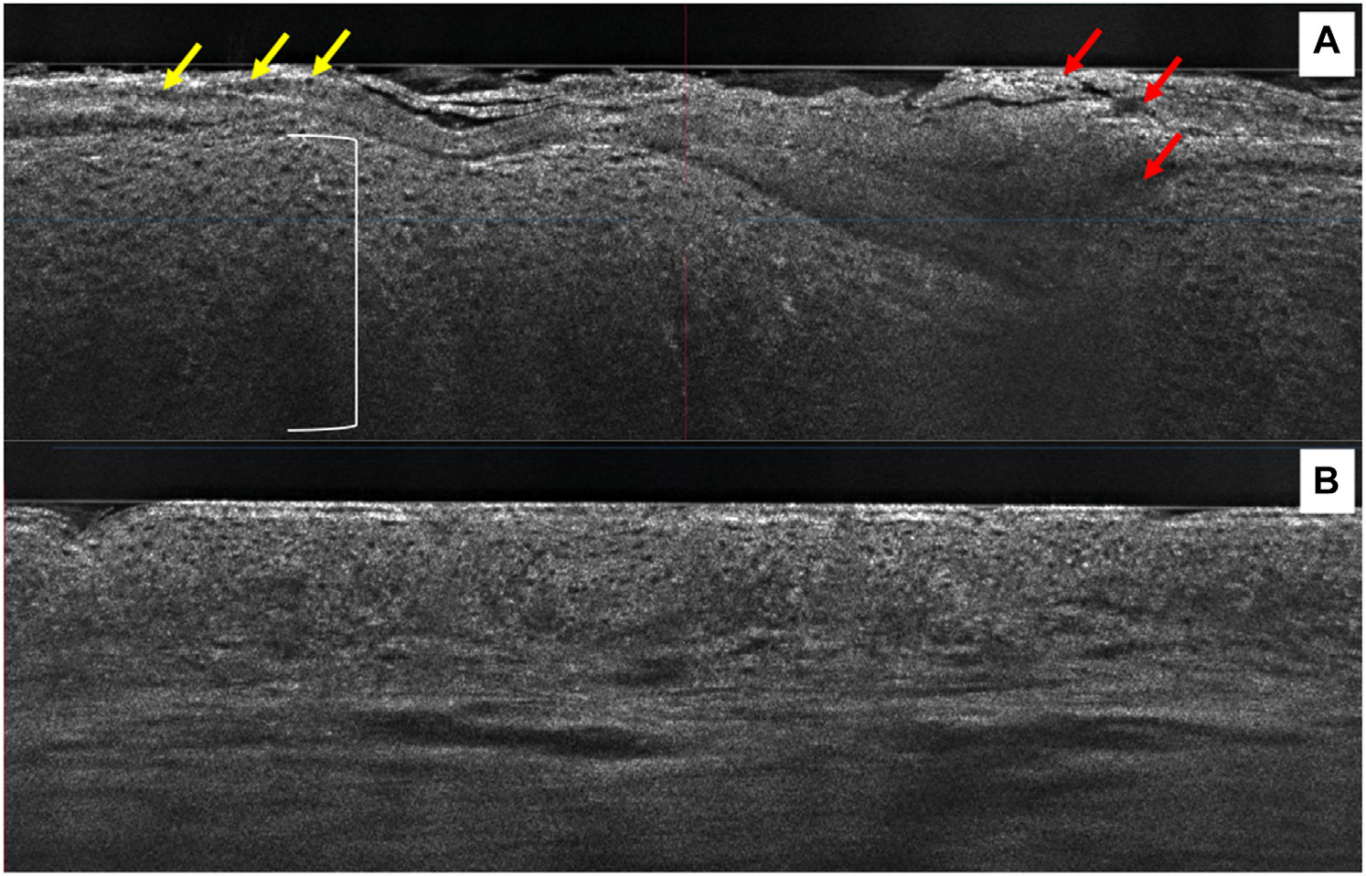
Two-dimensional vertical views. **A,** A psoriasis plaque showing classic psoriasiform hyperplasia (*white bracket*), with parakeratosis (*yellow arrows*) and bright small cells, likely neutrophils, clustered (neutrophilic lakes) in the stratum corneum as (*red arrows*). **B,** Adjacent lesion-free skin of the scalp.

**Fig 3:**
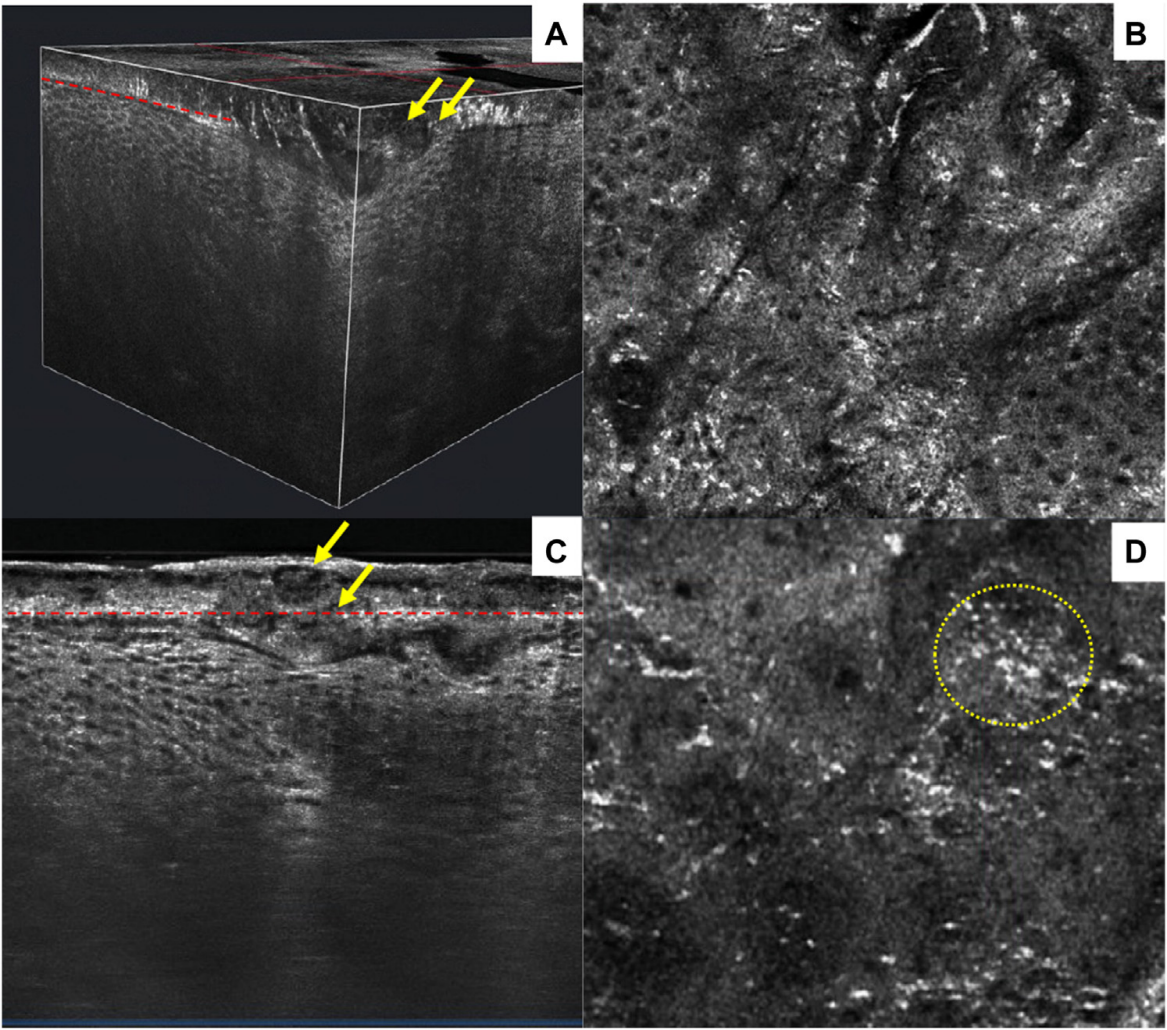
Multiple views of the psoriasis plaque using line-field confocal optical coherence tomography. **A,** Three-dimensional block view demonstrating epidermal hyperplasia with spongiform pustules of Kogoj (*yellow arrows*). The red dashed line indicates a transverse cut represented in image (**B**) showing papillomatosis. **C,** Collections of neutrophils within the stratum corneum (*yellow arrows*). The red dash line shows the en face view (**D**) of clusters of small bright cells within the stratum corneum (*yellow dotted circle*).

The lesion showed psoriasiform epidermal hyperplasia along with a papillomatous appearance on both vertical and horizontal sections (Figs 2 and 3). In addition, parakeratosis was present along with clusters of bright small white cells within the stratum corneum, likely correlating to collections of neutrophils. Small round areas filled with amorphous material were present at the spinous layer, which likely correlate to spongiform pustules of Kogoj, which were visible from the vertical view (Fig 3). Video 1, available on https://www.jaad.org.

## Histologic diagnosis

**Fig 4:**
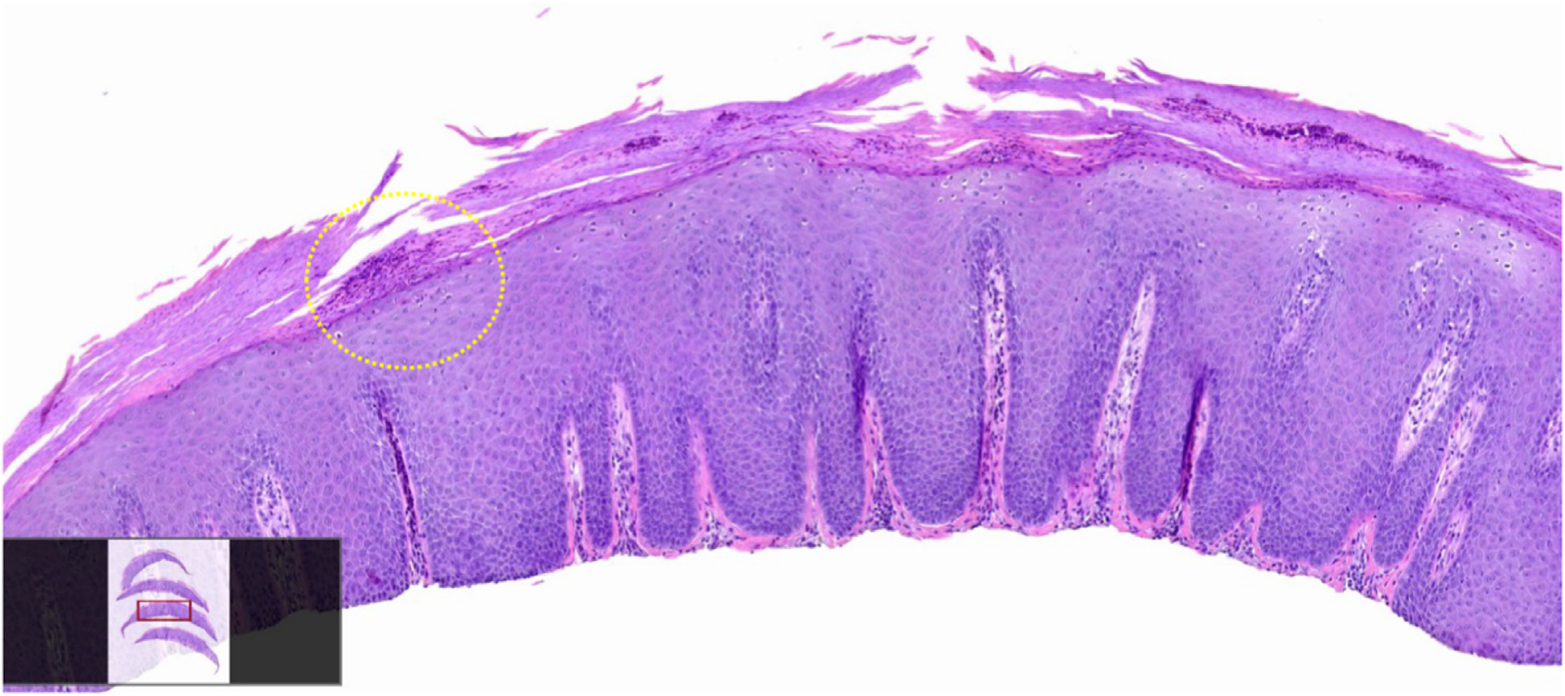
Histology shows that regular psoriasiform hyperplasia is present with parakeratosis and focal neutrophilic collections within the stratum corneum (*yellow dotted circle*).

Histology confirmed the diagnosis of psoriasis, showing regular psoriasiform epidermal hyperplasia, parakeratosis, and numerous collections of neutrophils within the stratum corneum (Fig 4).

## Key message

LC-OCT is a noninvasive, in vivo, high-resolution imaging technique that provides information regarding the epidermal and superficial to mid-dermal architecture. LC-OCT captures both vertical and horizontal 2-dimensional images to create a 3-dimensional image block to provide an image of the target lesion in real time. LC-OCT has a higher resolution than that of optical coherence tomography (5 μm vs 20-25 μm) and a higher penetration depth than that of reflectance confocal microscopy (>400 μm vs >200 μm).^1^ LC-OCT has been used to evaluate histopathologic features of skin cancer and some inflammatory skin conditions.^2^

Scalp psoriasis (SP) is an immune-mediated chronic inflammatory condition of the head and neck characterized by painful and pruritic erythematous thickened plaques.^3^^,^^4^ Although these lesions may closely mimic other inflammatory conditions, such as seborrheic dermatitis and atopic dermatitis, rapid histopathologic visualizations with LC-OCT may help confirm the diagnosis. LC-OCT imaging showed good histopathologic correlation with visualization of parakeratosis, clubbed and elongated rete ridges, and spongiform micropustules. Features such as follicular plugging and lymphocytic exocytosis are more characteristic of seborrheic dermatitis, and were not observed in this patient.^5^ This case report supports the use of LC-OCT imaging as a diagnostic aid for an earlier recognition of SP to guide clinical decision making and prevent longer-term complications, such as alopecia.

## Conflicts of interest

None disclosed.
